# Moesin expression by tumor cells is an unfavorable prognostic biomarker for oral cancer

**DOI:** 10.1186/s12885-017-3914-0

**Published:** 2018-01-08

**Authors:** Francisco Bárbara Abreu Barros, Agnes Assao, Natália Galvão Garcia, Suely Nonogaki, André Lopes Carvalho, Fernando Augusto Soares, Luiz Paulo Kowalski, Denise Tostes Oliveira

**Affiliations:** 1 Department of Stomatology, Area of Pathology, Bauru School of Dentistry, University of São Paulo, Alameda Octávio Pinheiro Brisolla, 9-75, Bauru, São Paulo, 17012-901 Brazil; 2 Adolfo Lutz Institute, Pathology Division, São Paulo, Brazil; 3 Fundação Pio XII Institution – Cancer Hospital of Barretos, Barretos, São Paulo, Brazil; 4 Rede D’Or Hospitals Network - Pathology Division, São Paulo, Brazil; 5 Department of Head and Neck Surgery and Otorhinolaringology, A.C.Camargo Cancer Center Hospital, São Paulo, Brazil

**Keywords:** Oral cancer, Squamous cell carcinoma, Podoplanin, Moesin, Biomarkers

## Abstract

**Background:**

Moesin is a member of the ERM (ezrin, radixin and moesin) proteins that participate in cell migration and tumor invasion through transductional signals sent to actin filaments by glycoproteins, such as podoplanin.

**Methods:**

This study aimed to evaluate the participation of moesin and podoplanin in the invasive tumor front of oral squamous cell carcinomas, and their influence on patients’ prognosis. Podoplanin and moesin immunoexpressions were evaluated by a semi-quantitative score method, based on the capture of 10 microscopic fields, at 400X magnification, in the invasive tumor front of oral squamous cell carcinomas. The association of moesin and podoplanin expression with clinicopathological variables was analyzed by the chi-square, or Fisher’s exact test. The 5 and 10 years survival rates were calculated by the Kaplan-Meier method and the survival curves were compared by using the log-rank test.

**Results:**

The immunohistochemical expression of moesin in the invasive front of oral squamous cell carcinomas was predominantly strong, homogenously distributed on the membrane and in the cytoplasm of tumor cells. The expression of moesin was not associated with clinical, demographic and microscopic features of the patients. Otherwise, podoplanin expression by malignant epithelial cells was predominantly strong and significantly associated with radiotherapy (*p* = 0.004), muscular invasion (*p* = 0.006) and lymph node involvement (*p* = 0.013). Strong moesin expression was considered an unfavorable prognostic factor for patients with oral squamous cell carcinomas, clinical stage II and III (*p* = 0.024).

**Conclusions:**

These results suggested that strong moesin expression by malignant cells may help to determine patients with oral squamous cell carcinoma and poor prognosis.

## Background

Moesin is a member of the ERM (ezrin, radixin and moesin) family of proteins that plays a role in cellular morphology, cell adhesion, controlling adherent junctions and cell motility, the key events of the carcinogenesis processes [1–3]. Specifically, the metastatic process involves moesin and other ERM proteins, leading to changes in cell morphology, cell to cell adhesion and in actin filament reorganization [4].

Recently, the overexpression of moesin in tumors has been correlated with metastasis and poor prognosis for the patient [5–8], including those with oral squamous cell carcinomas [9, 10]. Moesin probably participates in necessary conformational changes for an appropriate cell configuration, flexible enough to allow extravasation [11]. These processes occur through the phosphorylation of the C-terminal domain of moesin, which binds to actin filaments [12]. In the other domain, N-terminal, transmembrane molecules, such as podoplanin, are able to activate it, consequently inducing Rho phosphorylation. These downstream signals result in loss of adhesion, motility and higher rates of cell proliferation [13].

Podoplanin is a transmembrane glycoprotein and its overexpression has been associated with enhanced cancer cell motility, tumor invasion and poor patient prognosis in head and neck tumors [14–21]. The role of podoplanin in the tumor invasion process was first hypothesized by Martin-Villar et al. (2005), who reported the activation of ERM proteins by podoplanin through Rho-A phosphorylation. This connection is responsible for the maintanance of the ERM proteins in an open and active state conformation, strengthening the anchorage of podoplanin to cytoskeleton filaments.

To gain further understanding of the participation of moesin in tumor invasion process and its association with podoplanin in this pathway, for the first time, we analyzed the immunohistochemical association of moesin and podoplanin in oral squamous cell carcinomas with clinicopathological features and patients’ prognosis.

## Methods

This study was based on the analysis of eighty-four surgical specimens of patients who underwent surgical treatment for primary oral squamous cell carcinoma (OSCC) at the Head and Neck Surgery and Otorhinolaryngology Department of the A.C. Camargo Cancer Hospital, São Paulo, Brazil, from 1963 to 2012. Tumors were selected according to the following inclusion criteria: (1) oral squamous cell carcinoma located in the tongue, floor of the mouth, inferior gingiva and retromolar area, confirmed by biopsy; (2) patients not submitted to other previous treatment; (3) complete clinical data and follow up; (4) tumor tissue available for microscopic analysis. Clinical data were obtained from the medical records of the A.C. Camargo Cancer Hospital and included age, ethnic group, gender, tobacco and alcohol consumption, TNM stage (UICC: union for international cancer control, 2004), treatment (surgery, post-operative adjuvant radiotherapy), localization of the tumor and clinical follow-up (local recurrence, regional recurrence and death). Histopathological analysis included the following variables: vascular embolization, perineural infiltration, muscular infiltration, bone infiltration and lymph node involvement (pN+). Additionally, the histopathological grade of malignancy of oral squamous cell carcinomas was determined in hematoxylin & eosin stained sections [22].

### Podoplanin and moesin immunoexpression in oral squamous cell carcinomas

The immunohistochemistry technique followed the protocol of the Department of Pathology of the A.C. Camargo Cancer Hospital used previously by Faustino et al. (2008). The tumors sections were incubated with following primary monoclonal anti-podoplanin antibody: D2-40 clone, Dako North America Inc., M3619, Glostrup, Denmark, dilution 1:200 and with anti-moesin antibody 38/87 clone, Neomarkers, USA, dilution 1:400. Palatine tonsil was used as positive control for anti-podoplanin antibody and placenta for anti-moesin antibody. The lymphatic vessels were used as internal control.

### Immunohistochemistry evaluation

Approximately, 10 microscopic fields of each tumor specimen were captured with a digital camera (AxiocamMRc, Zeiss) attached to a microscope (Axioskop 2 Plus, Zeiss), at 400X magnification to evaluate the immunoexpression of podoplanin and moesin. Images of each tumor field were sequentially captured in the invasive tumor front and recorded in a computer program system (Axiovision 4.9, Zeiss, Jena, Germany).

Two experienced pathologists evaluated podoplanin and moesin expressions by malignant cells, based on a semi-quantitative score system, previously established by Faustino et al. (2008). The final score was determined by the sum of the immunostaining intensity and the percentage of positive immunostaining cells. Subsequently, the oral squamous cell carcinomas were classified into 3 groups: 0 = absent immunostaining; 1 = weak immunostaining; 2 = strong immunostaining [23].

### Statistical analyses

The statistical analyses were performed using SPSS Statistical software version 21.0 (SPSS Inc., Chicago, IL, USA). The association of podoplanin and moesin immunoexpression by neoplastic cells with the clincopathological variables was verified by the Chi-square (*x*^2^), or Fischer’s exact tests. For these analyses, the absent and weak immunoexpressions were grouped together, obtaining a final group of absent/weak tumor immunoexpression and strong tumor immunoexpression. The overall survival probability in 5 and 10 years was estimated using the Kaplan-Meier method, and survival curves were compared by log-rank test. The follow-up period considered for overall survival consisted of the time between the date of surgery and death or the date of the last information about the patient. Cox regression analysis of the survival data was performed to test any statistical significance of regression coefficients. For all statistical analyses applied, *p* values of less than 0.05 were considered statistically significant.

## Results

For the present study, the sample consisted of 84 patients with oral squamous cell carcinomas, predominantly male (85.7%), white (95.2%), with ages varying from 33 to 95 years (mean of 58 years). According to risk factors for OSCC, alcohol (67.9%) and tobacco (84.5%) consumption were reported by the majority of the patients. The most common location for OSCC was the tongue (54.8%), followed by floor of the mouth (28.6%). Moreover, most patients were clinically classified as T2 (59.5%) or T3 (40.5%), and N+ (48.8%), at the time of physical examination.

Only three patients (3.6%) were not submitted to elective neck dissection, 78.6% were submitted to ipsilateral neck dissection and 17.8% were dissected bilaterally. Thirty-eight patients (45.2%) who were submitted to neck dissection were positive for lymph node metastasis (pN+), shown by histopathological analysis.

Local recurrence occurred in 25% of the patients, while regional recurrence was present in 16.7% and local and regional recurrences, simultaneously, occurred in 6% of the patients.

Most tumors, 86.9% were classified as well/moderately differentiated; and 13.1% were classified as poorly differentiated, according to the histopathological grade of tumor malignancy [22].

### Immunohistochemical expression of moesin in oral squamous cell carcinomas

**Fig. 1:**
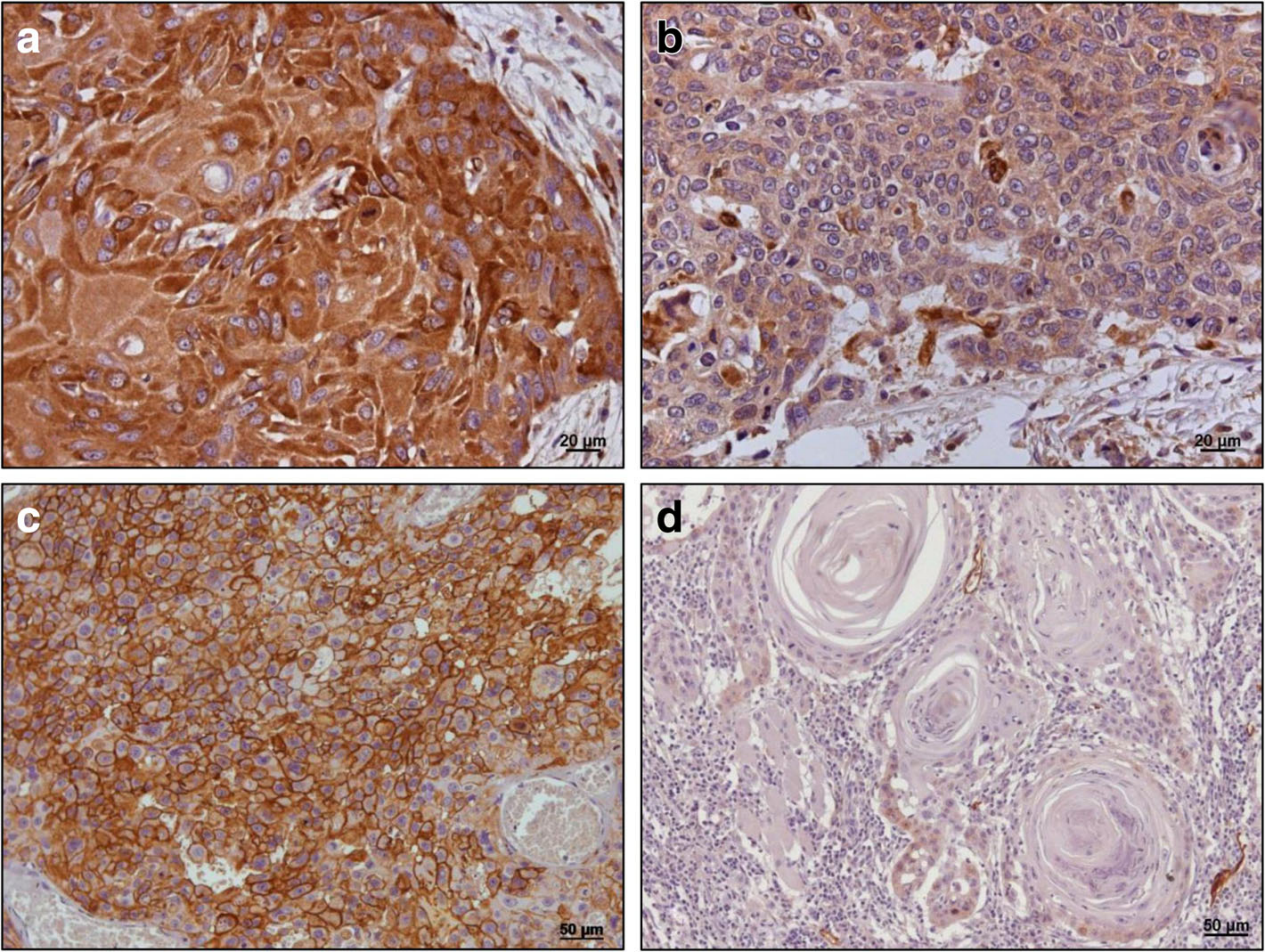
Immunohistochemical expression of moesin in oral squamous cell carcinomas. Strong (**a**) and weak cytoplasmic (**b**) moesin expression (**a**, IHQ 400X; (**b**, IHQ 200X). Membranous strong (**c**) and weak/absent podoplanin expression (**d**) (**c**, IHQ 200X; **d**, IHQ 200X)

Immunohistochemical expression of moesin in the invasive front of oral squamous cell carcinomas was weak in 40 tumors, while in 44 tumors the expression was strong. A predominantly cytoplasmic moesin expression was observed in most of the tumors. The keratin pearls and some areas with more differentiated neoplastic cells showed weak/negative moesin immunoexpression (Fig. 1).

Clinical, demographic and microscopic features analyzed were not statistically associated with moesin expression (Tables 1 and 2).

**Table 1:** Expression of podoplanin and moesin at invasive tumor front of 84 oral squamous cell carcinoma, according to clinical data and follow-up. A.C. Camargo Cancer Hospital, São Paulo, Brazil, 1963 to 2012

Variable		Moesin				*p*	Podoplanin				*p*
		Weak		Strong			Weak		Strong		
		N	%	N	%		N	%	N	%	
Gender											
Male		35	87.5	37	84.1	0.656	45	91.8	27	77.1	0.058
Female		5	12.5	7	15.9		4	8.2	8	22.9	
Age											
≤ 58 years		22	55	20	45.5	0.382	24	49	18	51.4	0.825
> 58 years		18	45	24	54.5		25	51	17	48.6	
White		39	97,5	41	93.2	0.618	46	93.9	34	97.1	0.637
Non-white		1	2,5	3	6.8		3	6.1	1	2.9	
Tobacco^a^											
Yes		31	93.9	40	95.2	0.999	43	95.6	28	93.3	0.999
No		2	6.1	2	4.8		2	4.4	2	6.7	
Alcohol^a^											
Yes		26	76.5	31	73.8	0.790	32	71.1	25	80.6	0.346
No		8	23.5	11	26.2		13	28.9	6	19.4	
T Stage											
T2		25	62.5	25	56.8	0.596	27	55.1	23	65.7	0.329
T3		15	37.5	19	43.2		22	44.9	12	34.3	
N Stage											
N0		22	55	21	47.7	0.505	24	49	19	54.3	0.631
N+		18	45	23	52.3		25	51	16	45.7	
Lymph Node Involvement											
No		2	5	1	2.3	0.679	1	2	2	5.7	0.669
Ipsilateral		30	75	36	81.8		39	79.6	27	77.1	
Contralateral		8	20	7	15.9		9	18.4	6	17.1	
Radiotherapy											
Yes		20	50	17	38.6	0.295	21	42.9	26	74.3	***0.004***
No		20	50	27	61.4		28	57.1	9	25.7	
Recurrence											
Yes		22	55	21	47.7	0.505	26	53.1	15	42.9	0.356
No		18	45	23	52.3		23	46.9	20	57.1	
Second Tumor											
Yes		10	25	4	9.1	0.061	6	12.2	8	22.9	0.198
No		30	75	40	90.0		43	87.8	27	77.1	
TOTAL		40	100	44	100		36	100	55	100	

N: Number of tumors; *p*: value obtained by chi-squares test or Fischer’s exact test

^a^Excluded patients with lost records

*p*<0.05 was considered statistically significant

**Table 2:** Immunohistochemical distribution of moesin and podoplanin at invasive front tumor of 84 oral squamous cell carcinoma, according to microscopical variables. A.C. Camargo Cancer Hospital, São Paulo, Brazil, 1963 to 2012

Variable	Moesin				*p*	Podoplanin				*p*
	Weak		Strong			Weak		Strong		
	N	%	N	%		N	%	N	%	
Vascular Embolization										
Yes	25	64.5	28	63.6	0.914	35	71.4	18	51.4	0.061
No	15	37.5	16	36.4		14	28.6	17	48.6	
Perineural Infiltration										
Yes	28	70	29	65.9	0.688	34	69.4	23	65.7	0.722
No	12	30	15	43.1		15	30.6	12	34.3	
Muscular Infiltration										
Yes	31	77.5	38	86.4	0.289	45	91.8	24	68.6	***0.006***
No	9	22.5	6	13.6		4	8.2	11	31.4	
Bone Infiltration										
Yes	2	5	3	6.8	0.999	1	2	4	11.4	0.155
No	38	95	41	93.2		48	98	31	88.6	
Lymph node Involvement^a^										
pN0	21	55.3	22	51.2	0.712	20	41.7	23	69.7	***0.013***
pN+	17	44.7	21	48.8		28	58.3	10	30.3	
TOTAL	40	100	44	100		49	100	35	100	

N: Number of tumors; *p*: value obtained by chi-squares test or Fischer’s exact test. pN0: patients without node metastasis; pN+: patients with node metastasis

^a^Excluded patients who were not submitted to elective neck dissection

*p*<0.05 was considered statistically significant

### Immunohistochemical expression of podoplanin in oral squamous cell carcinomas

The expression of podoplanin in oral squamous cell carcinomas was weak in 49 tumors and strong in 35 tumors. Membranous and cytoplasmic podoplanin expression was observed in oral squamous cell carcinomas with a predominance of the membranous expression (Fig. 1).

The strong podoplanin expression was associated with post-operative radiotherapy (*p* = 0.004) in those patients diagnosed with squamous cell carcinomas. Podoplanin expression was not associated with the demographic and clinical features analyzed (Table 1).

Concerning microscopic features analyzed, podoplanin expression in patients with muscle infiltration was weak compared with those without infiltration (*p* = 0.006). Moreover, the podoplanin expression was significantly associated with lymph node metastasis (pN+). In other words, most patients with lymph node metastasis presented weak expression of podoplanin (*p* = 0.013), as illustrated in Table 2.

Overall analysis of podoplanin and moesin expression in oral squamous cell carcinomas was not statistically associated (*p* = 0.460).

### Overall survival analysis

**Fig. 2:**
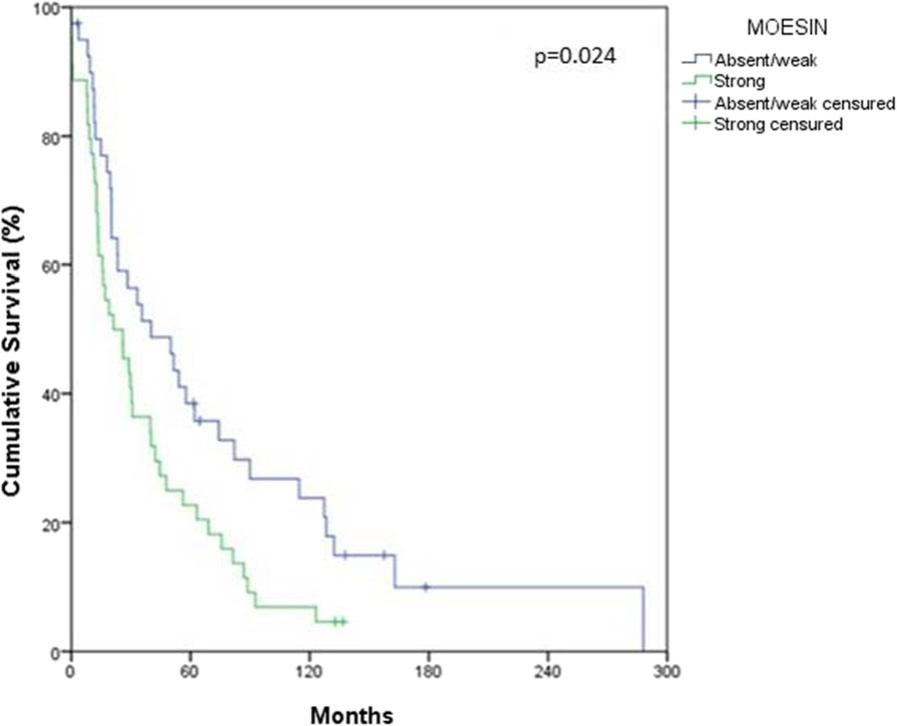
Prognostic value for moesin expression in oral squamous cell carcinomas. Cumulative overall survival rates by Kaplan-Meier method. (*p* = 0.024)

Overall survival rates varied from 0.01 to 288 months, mean of 57.2 months. Only moesin expression showed statistically significant differences (*p* = 0.024). The overall survival rate, in 5 years and 10 years, for patients with oral squamous cell carcinomas and strong moesin expression was reduced from 38.5%, to 23.8%, respectively. For oral squamous cell carcinomas in patients with weak moesin expression the survival rate varied from 22.7% in 5 years to 6.8% in 10 years. These differences in oral cancer patients’ survival rates for moesin expression were statistically significant (Table 3 and Fig. 2).

**Table 3:** Overall survival rates in 5 and 10 years of 84 patients with oral squamous cell carcinoma according to demographic features, risk factors, lymph node metastasis (pN), moesin and podoplanin, expression. A.C. Camargo Cancer Center, São Paulo, Brazil, 1963 to 2012

Variable	Overall Survival		*p*
	5 years (%)	10 years (%)	
Gender			
Male	33.8	15.4	0.145
Female	8.3	8.3	
Age			
≤ 58 years	38.1	19.3	0.075
> 58 years	22	9.8	
Tobacco			
Yes	28.6	11.2	0.282
No	25	25	
Alcohol			
Yes	25	9.6	0.512
No	31.6	12.6	
Radioterapy			
Yes	32.4	11.8	0.802
No	28.4	16.5	
Muscle Infiltration			
Yes	30.9	11.5	0.313
No	26.7	26.7	
Pn			
pN0	39.5	21.2	0.081
pN+	21.7	8.1	
Moesin			
Weak	38.5	23.8	***0.024***
Strong	22.7	6.8	
Podoplanin			
Weak	27.1	5.0	0.133
Strong	34.3	25.7	

*pN+: Histopathological lymph node metastasis; pN0: Absence of lymph node metastasis, histopathologically

*p* = value obtained by log-rank test

*p*<0.05 was considered statistically significant

Based on Cox regression analysis, patients with oral squamous cell carcinomas and strong moesin expression by neoplastic epithelial cells had 1.737-fold higher chance of relative risk of death (*p* = 0.022), as illustrated in Table 4.

**Table 4:** Moesin expression analysis by Cox proportional hazard model. A.C. Camargo Cancer Hospital,São Paulo, Brazil, 1963 to 2012

Variable	Overall Survival		
		*p*	HR (95%CI)
Moesin	Weak (0)	0.022	1.737 (1.083- 2.788)
	Strong (1)		

HR: Hazard according to gender, age, lymph node metastasis, radiotherapy, muscle infiltration, tobacco, alcohol, moesin and podoplanin expression by neoplastic cells. CI: Confidence Interval

## Discussion

To date, the intracellular location of moesin during oral carcinogenesis has been poorly understood. Moesin translocation from plasma membrane to the cytoplasm of neoplastic cells has been demonstrated in a previous study [24] and it may reduce the ability to form cell-cell contacts, as well as, influence the cytoskeleton remodeling and tumor invasion process, when overexpressed in the cytoplasm [24]. Furthermore, podoplanin linkage with the N-terminal moesin domain activates the ERM proteins, consequently inducing Rho-A phosphorylation and in the maintenance of the ERM proteins in an active form (open conformation), in the cytoplasm [25].

The moesin immunoprofile in the invasive front of oral squamous cell carcinomas, clinical stages II and III, was predominantly cytoplasmic and strong. Keratin pearls and some areas with more differentiated neoplastic cells showed negative moesin expression. Confirming these results, other authors described the membranous and cytoplasmic moesin expression in oral squamous cell carcinomas, [5, 9, 10]. Belbin et al. (2005) showed that membranous and cytoplasmic moesin expression increased when normal epithelium was compared with dysplastic epithelium and/or with tumor samples [10]. The authors affirmed that moesin expression was significantly associated with head and neck squamous cell carcinomas progression. In addition, as found in the present study, the loss of moesin expression in more differentiated cells was found in keratin pearls [5, 26].

The expression of moesin was not associated with the clinical, demographic and microscopic features analyzed (*p* > 0.05), reinforcing previous findings [5, 7, 9, 26]. Kobayashi et al. (2004) found a significant association of moesin with the size of the tumor, but the sample analyzed varied from T1 to T4 tumors. Differently, the present study was composed of T2 and T3 tumors and no association could be found with moesin expression, as it was restricted to a limited size of tumors. According to our previous experiences with analyses of protein expression by oral neoplastic cells, the OSCCs clinically classified as T2 and T3 are better for verifying their influence on patients’ prognosis, than T1 (initial tumors) or T4 (advanced tumors).

Regarding podoplanin expression by tumor cells, as previously observed by de Vicente et al. (2015) and Tsuneki et al. (2013), the present results showed higher podoplanin expression in the earlier stages of tumors and in highly differentiated malignant cells. These studies [27–29] verified that podoplanin expression was inversely correlated with the degree of neoplastic epithelial cell differentiation. In agreement with the cited studies, the results found in the present study confirmed a weak/absent podoplanin expression in the majority of tumors with muscular infiltration and lymph node involvement (pN+).

Initial studies about podoplanin related strong podoplanin expression to the worst prognosis and more advanced stages of oral squamous cell carcinomas [14–16, 30, 31]. Instead, the present results showed the opposite side of those findings, and the authors suggest further studies should be conducted about podoplanin expression in less differentiated tumors to validate these findings.

Considering demographic and clinical features, the strong expression of podoplanin was observed mainly in those patients submitted to post-operative radiotherapy (*p* = 0.004) [14, 18, 19, 28, 30]; however, further studies are necessary for better evaluation of radiotherapy and podoplanin expression.

Concerning clinical stages T and N, our results failed to find an association between podoplanin expression and the clinical stages of the tumor, probably because we included only T2 and T3 tumors, while in other studies, the tumor stages varied from I to IV [14, 16, 19, 20, 30].

No statistically significant association (*p* = 0.460) was found between podoplanin and moesin immunoexpression by malignant cells in the invasive front of oral squamous cell carcinomas. As this was the first study about the joint expression of podoplanin and moesin in oral cancer, further investigations based on in vitro assays are necessary for better evaluation of the participation of these molecules together in the tumor invasion process.

The overall survival and the prognostic value of moesin and podoplanin expression in oral squamous cell carcinomas were analyzed in this study. Moesin expression was considered a significant prognostic factor for oral squamous cell carcinomas. The overall survival rate in 5 years was 22.7% for those patients with the strong moesin expression, while for patients with weaker moesin expression, the overall survival rates in 5 years was 38.5%. In ten years, overall survival rates for strong moesin expression were 6.8% and 23.8% for weak expression. Moreover, the patient with strong moesin expression presented a 1737 times higher risk of dying, compared with those with the weak expression. These results are consistent with those found by Kobayashi et al. (2004) and Schlecht et al. (2012) in oral squamous cell carcinomas and in breast cancer [6, 32].

Interestingly and corroborating the above results, a recent study conducted by Li et al. (2015) observed the knockdown of moesin in oral squamous cell carcinomas cell lines and a significantly reduction in migration and invasion [33]. Furthermore, moesin silencing showed an increase in cell-cell adhesion. By cell spreading assay, moesin inhibition reduced filopodia formation, indicating the role of moesin in cytoskeletal modifications. This study was in agreement with the present results, which indicated that the weak expression of moesin could be related to higher survival rates in oral squamous cell carcinoma patients.

Podoplanin expression, in turn, was not considered as a significant prognostic factor for oral squamous cell carcinomas, as related by de Vicente et al. (2015) and Dos Santos Almeida et al. (2013). Although previous results considered [14–16, 30, 31] that strong podoplanin expression was related to poor prognosis, we could not predict the prognostic significance of podoplanin in those tumors, probably because strong podoplanin expression was related to initial tumors in earlier stages of the neoplastic cell differentiation process [27–29]. Therefore, considering the latest controversial studies concerning podoplanin expression, we suggest that further studies with a larger sample should be conducted to confirm the potential prognostic value of this protein in oral squamous cell carcinomas.

## Conclusions

The weak immunohistochemical expression of moesin by neoplastic epithelial cells could be used as a favorable prognostic marker for oral squamous cell carcinomas. Additionally, the weaker expression of podoplanin was associated with lymph node involvement and muscular infiltration in oral squamous cell carcinomas, indicating that podoplanin expression occurs in more differentiated cells and at earlier stages of the tumors. Moreover, malignant cells of oral squamous cell carcinomas expressed both podoplanin and moesin, but these proteins probably act individually in tumor invasion process.

## Abbreviations

ERM: Ezrin, radixin, moesin
OSCC: Oral squamous cell carcinoma
SPSS: Statistical Package for the Social Sciences
TNM: Tumor size, lymph nodes and metastasis
UICC: Union for international cancer control
